# Supplementary material for: Regulation of mutant TERT by BRAF V600E/MAP kinase pathway through FOS/GABP in human cancer
Source: Nat Commun. 2018 Feb 8;9:579. doi: 10.1038/s41467-018-03033-1 (PMC5805723; doi:10.1038/s41467-018-03033-1)
Supplement: Supplementary file 1 — Supplementary Information [file 41467_2018_3033_MOESM1_ESM.pdf]

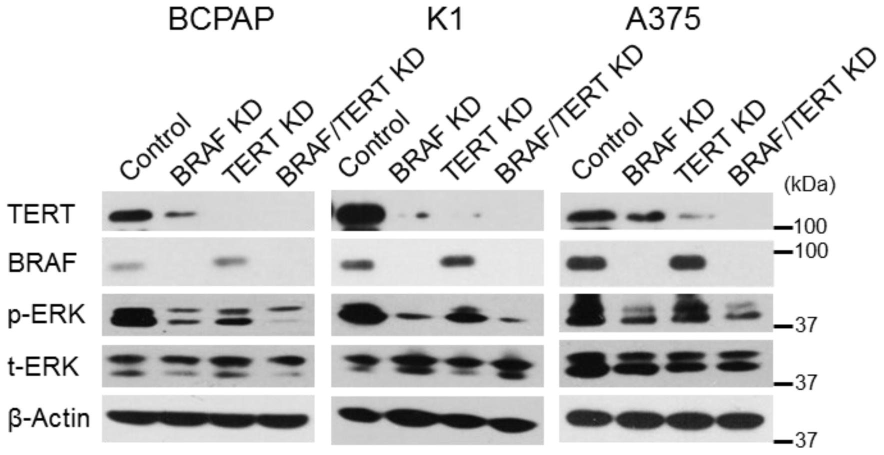

Supplementary Figure 1. Western blotting analyses of TERT, BRAF, phosphorylation of ERK (p-ERK), total ERK (t-ERK), and beta-Actin. Control scramble shRNA- or BRAF shRNA- expressing cells were co-transfected with control siRNA or TERT siRNA to also knockdown (KD) TERT.

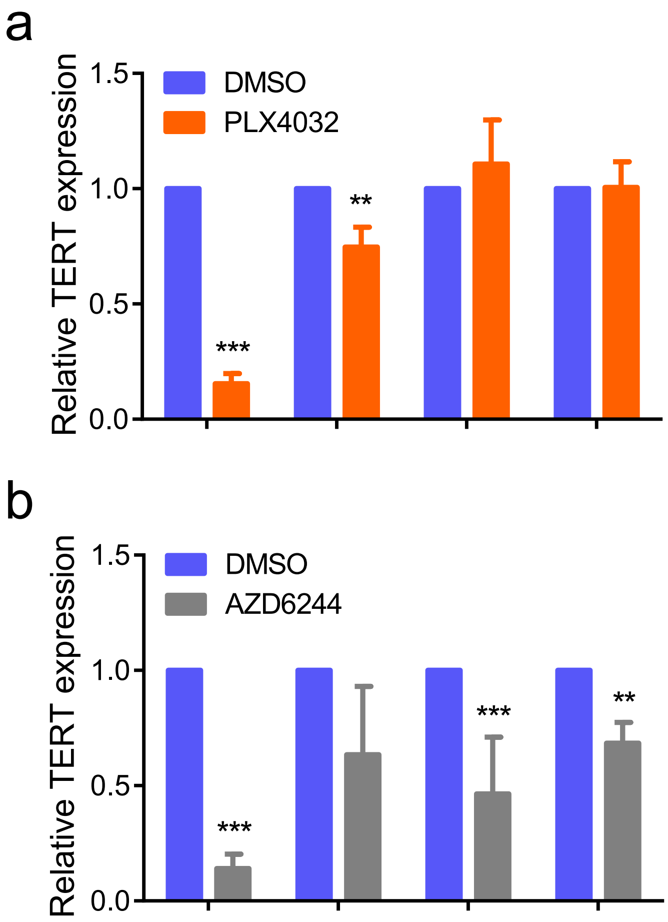

Supplementary Figure 2. Summarized effects of PLX4032 (A) and AZD6244 (B) on *TERT* mRNA expression. Each bar represents the average ± SD of the cells with the same genotypes as follows: BRAF-mut and TERT-mut cell lines: BCPAP, K1, OCUT1, A375, and M14; BRAF-mut and TERT-wt cell lines: SK-MEL-1, SK-MEL-3, and RKO; BRAF-wt and TERT-mut cell lines: TPC1, KAT18, C643, CHL-1, SK-MEL-2, and Mewo; BRAF-wt and TERT-wt cell lines: FB1, WRO, and HTORI3. Compared with the corresponding control of DMSO, **P<0.01, ***P<0.001, by independent *t*-test.

Figure 1e

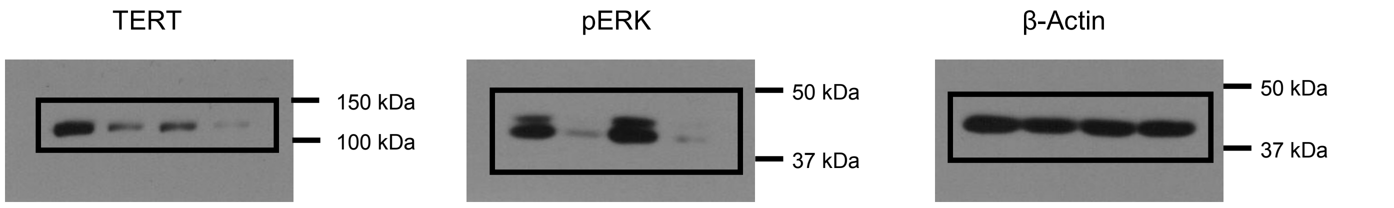

Figure 2a

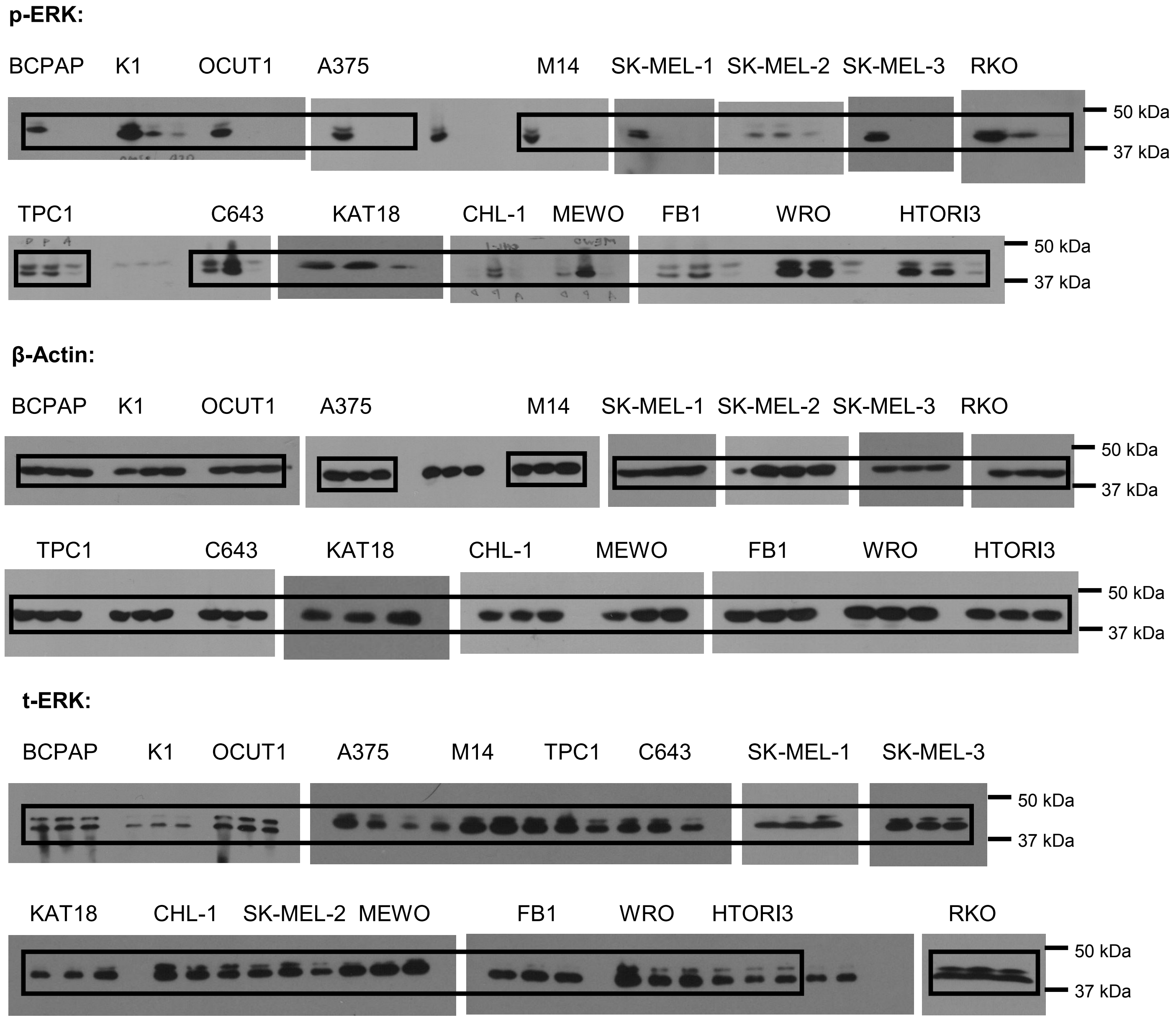

Supplementary Figure 3. Uncropped western blotting images for Figures 1e and 2a.

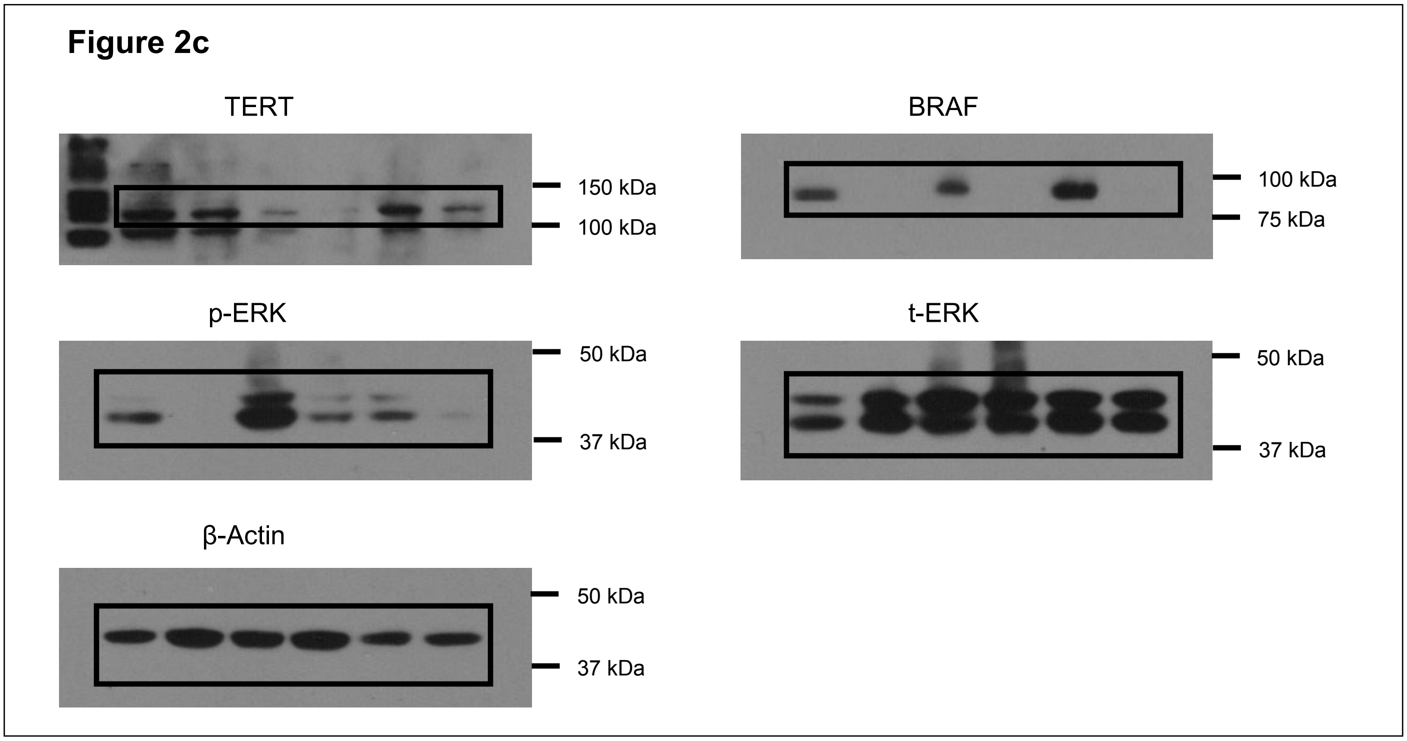

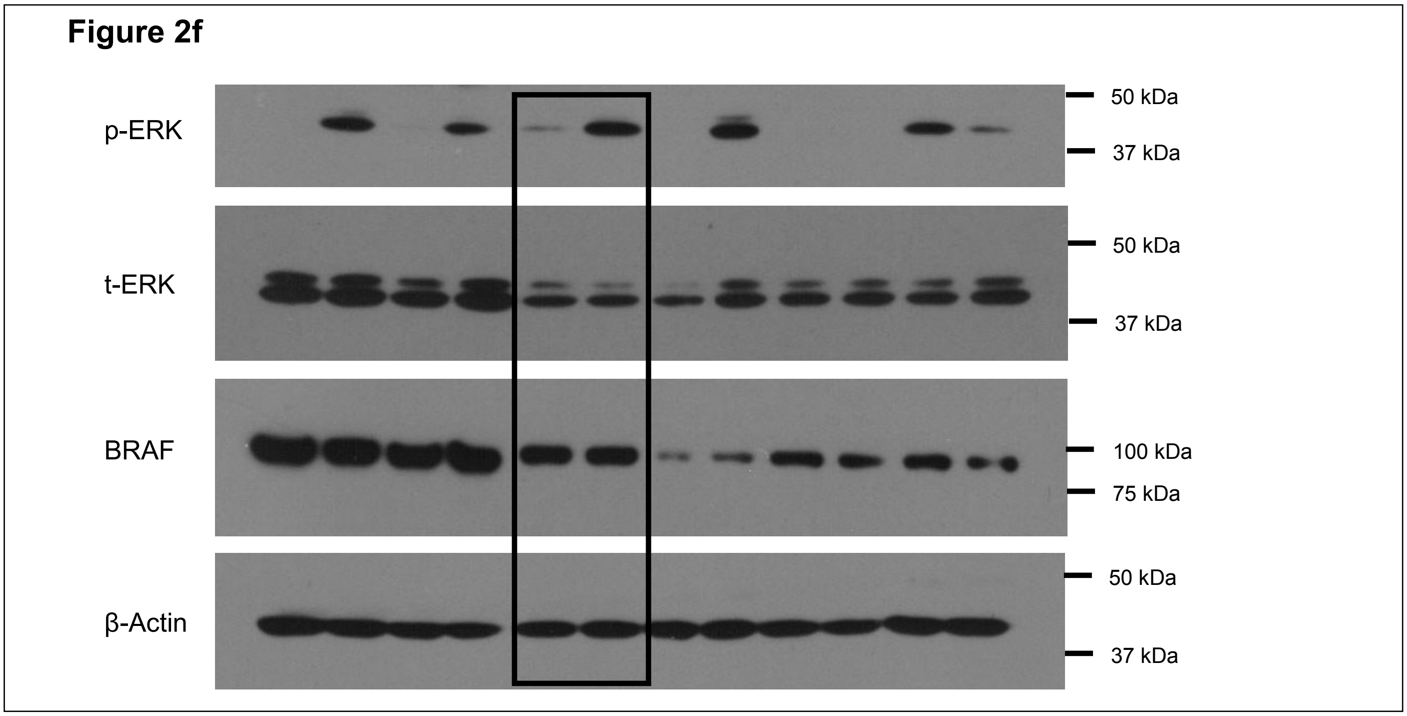

Supplementary Figure 4. Uncropped western blotting images for Figures 2c and 2f.

Figure 3a

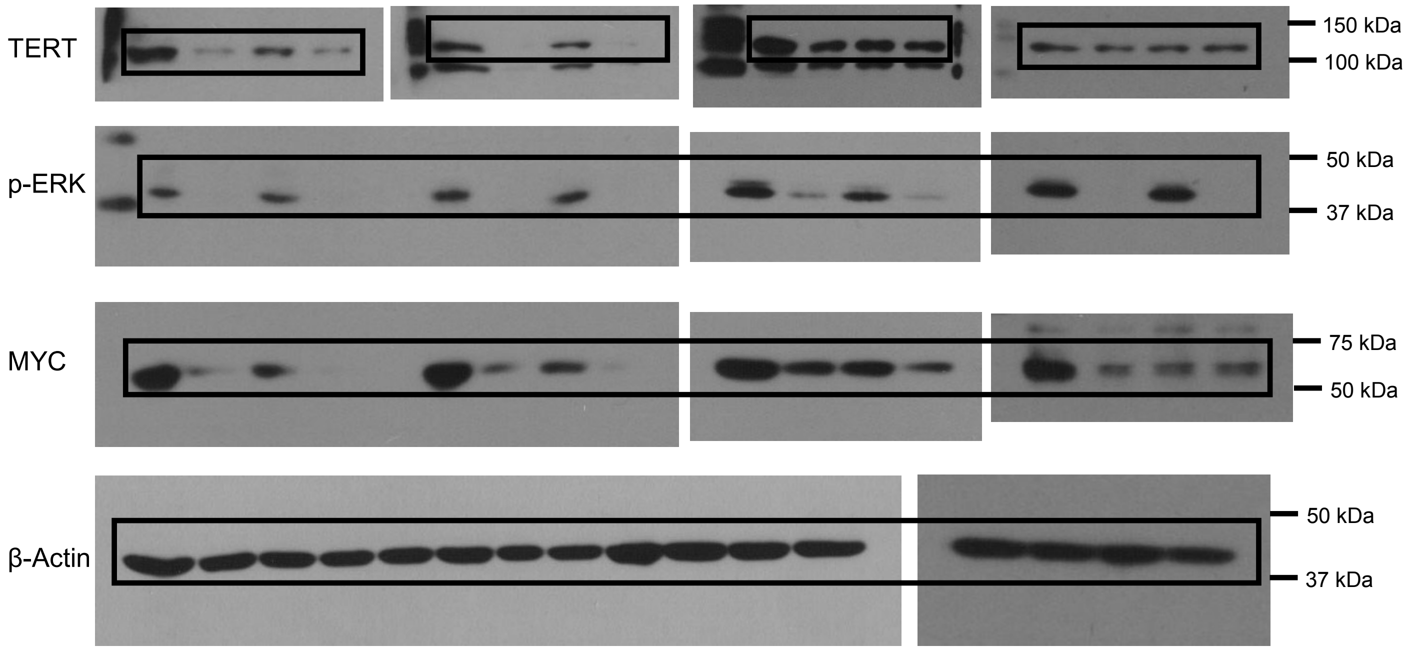

Figure 3b

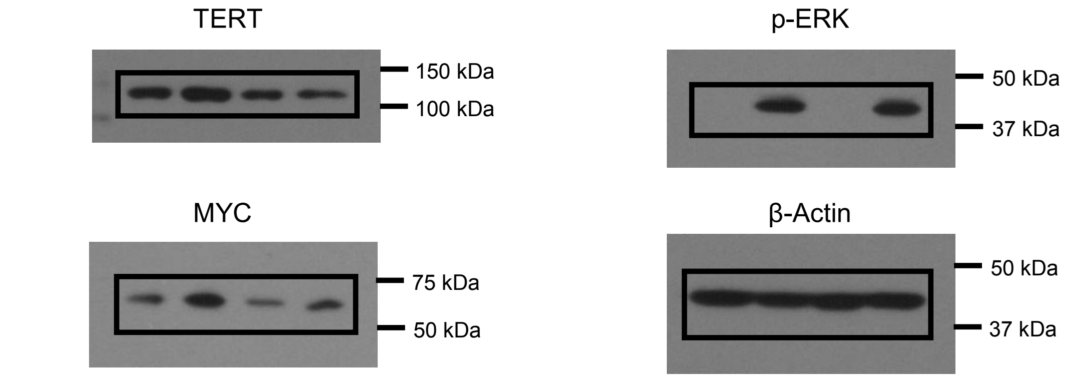

Figure 4b

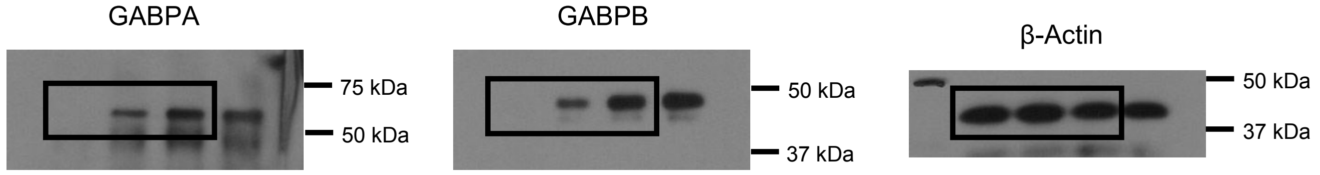

Figure 4d

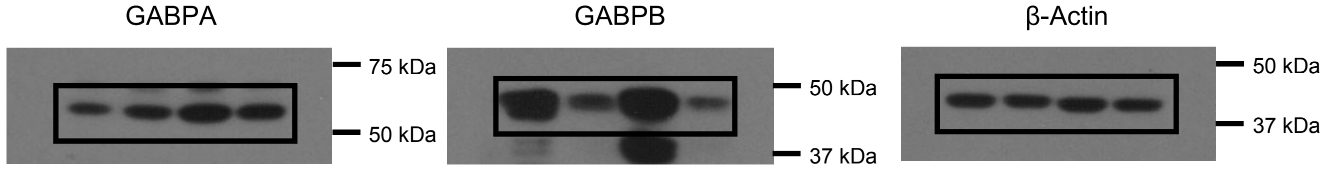

Supplementary Figure 5. Uncropped blots for Figures 3a, 3b, 4b, and 4d.

Figure 5c

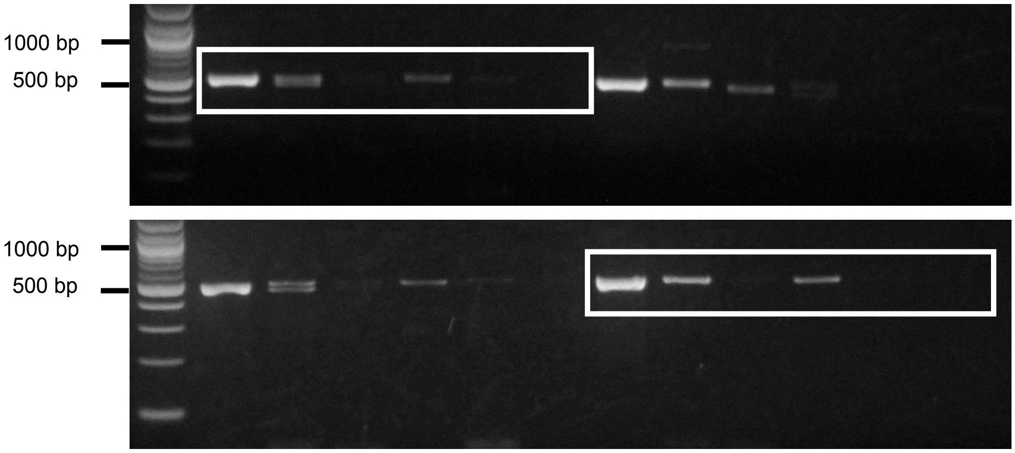

Figure 5d

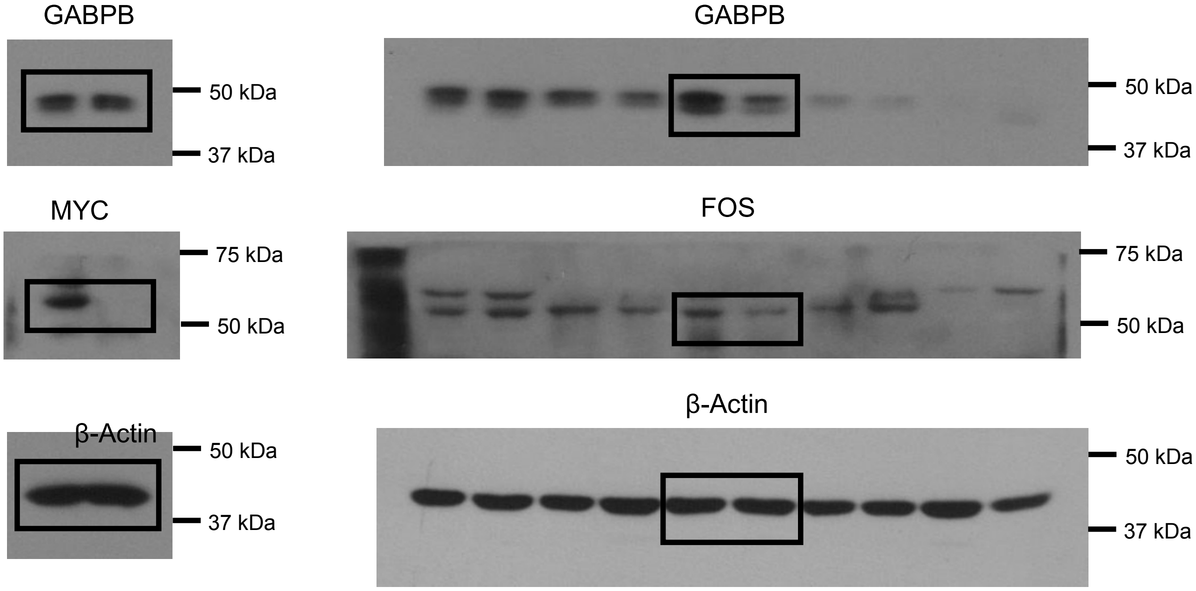

Figure 5f

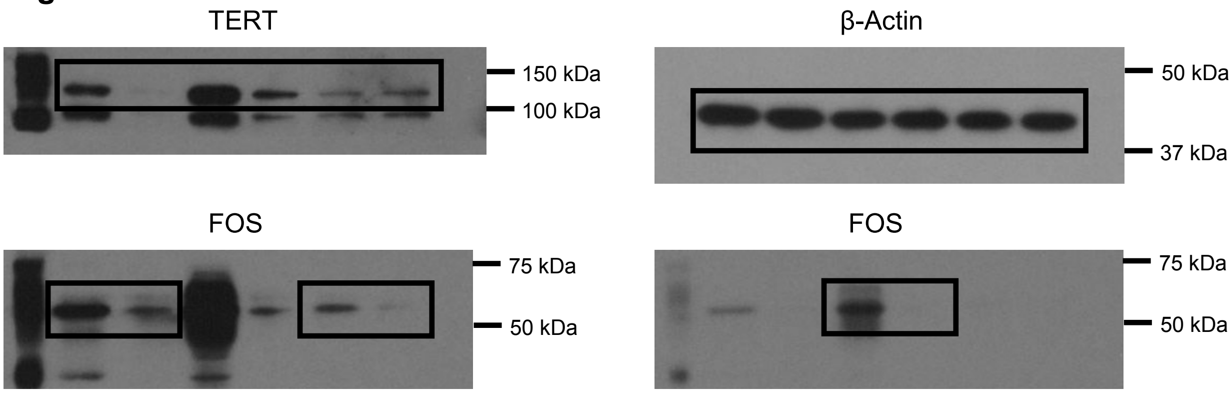

Supplementary Figure 6. Uncropped gel images for Figure 5c, and uncropped western blotting images for Figures 5d and 5f.

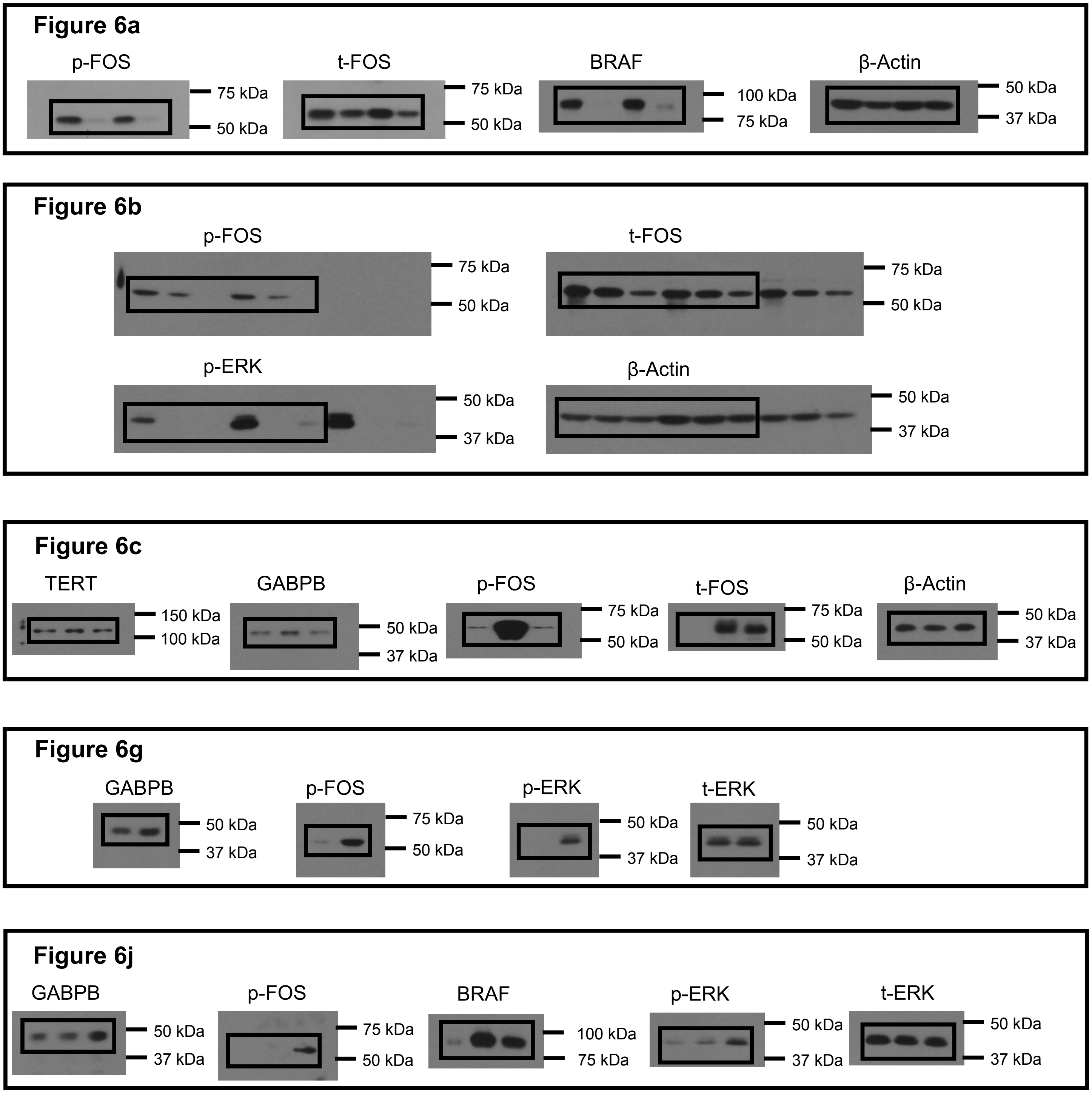

Supplementary Figure 7. Uncropped western blotting images for Figures 6a, 6b, 6c, 6g and 6j.

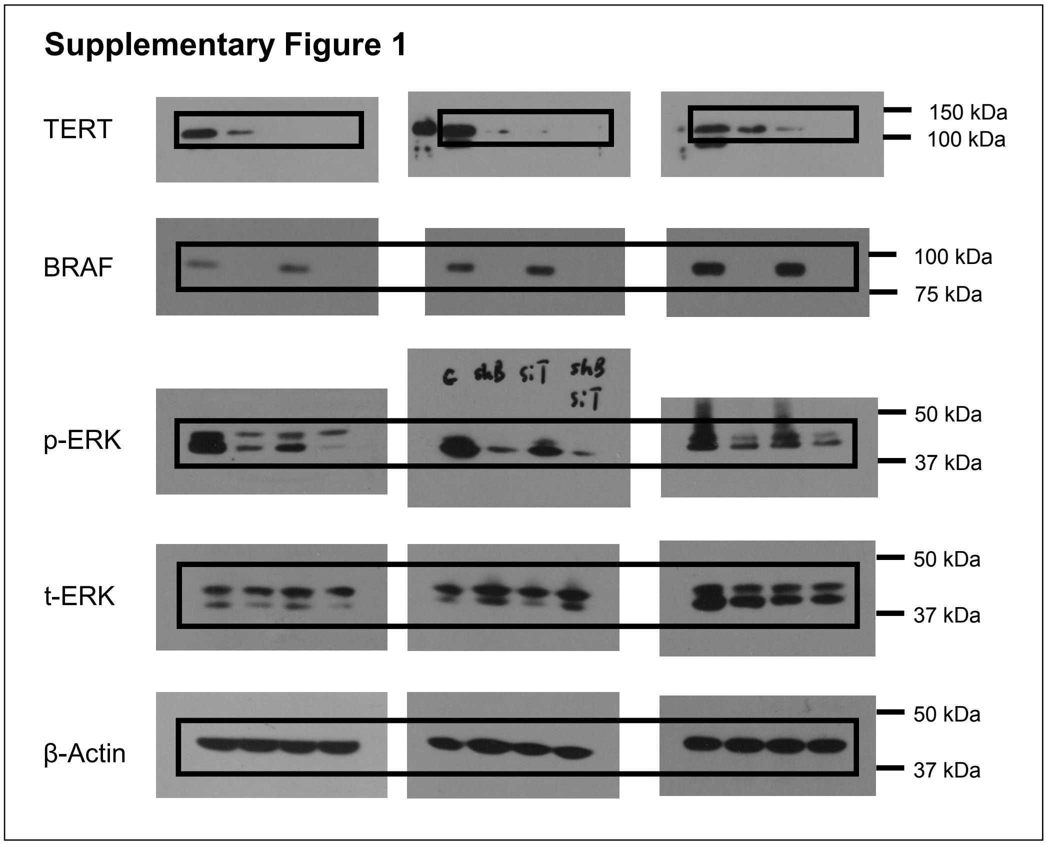

Supplementary Figure 8. Uncropped western blotting images for Supplementary Figure 1.

Supplementary Table 1. Genotypes of *BRAF* and *TERT* genes in various human cancer cell lines used in the present study

Cell line	BRAF	TERT promoter	Derivation
BCPAP	V600E, homozygous	C228T/C229T, heterozygous	thyroid carcinoma
K1	V600E, heterozygous	C228T, heterozygous	thyroid carcinoma
OCUT1	V600E, heterozygous	C250T, homozygous	thyroid carcinoma
TPC1	wildtype	C228T, heterozygous	thyroid carcinoma
KAT18	wildtype	C228T, heterozygous	thyroid carcinoma
C643	wildtype	C228T, heterozygous	thyroid carcinoma
FB1	wildtype	wildtype	thyroid carcinoma
WRO	wildtype	wildtype	thyroid carcinoma
HTORI3	wildtype	wildtype	thyroid epithelium
A375	V600E, homozygous	C250T, heterozygous	melanoma
M14	V600E, heterozygous	C250T, heterozygous	melanoma
SK-MEL-1	V600E, heterozygous	wildtype	melanoma
SK-MEL-3	V600E, heterozygous	wildtype	melanoma
SK-MEL-2	wildtype	C250T, homozygous	melanoma
MeWo	wildtype	C250T, heterozygous	melanoma
CHL-1	wildtype	C228T, heterozygous	melanoma
RKO	V600E, homozygous	wildtype	colon carcinoma
SW48	wildtype	wildtype	colon carcinoma

Supplementary Table 2. Primer sequences for qRT-PCR, luciferase reporters cloning, Mutagenesis, and ChIP assays used in the present study

Name	Primer Sequence 5'-3'
**qRT-PCR**	
TERT-F	5'-GTCCGAGGTGTCCCTGAGTA-3'
TERT-R	5'-CAGGGCCTCGTCTTCTACAG-3'
GAPDH-F	5'-TGCACCACCAACTGCTTAGC-3'
GAPDH-R	5'-GGCATGGACTGTGGTCATGAG-3'
**Luciferase reporter constructs**	
GABPA-F	5'-GGGGTACCACTGACCGGCCAAAGGTTAG-3'
GABPA-R	5'-CCCTCGAGTCGGAGGGGAGCTTGAACTA-3'
GABPB-F	5'-GGGGTACCTCCTTCCGTCTCCCAGGATT-3'
GABPB-R	5'-CCCTCGAGAATCCCCACCGAAAAGTCCC-3'
TERT-F	5'-CGACGCGTGGCTCCCAGTGGATTCGC-3'
TERT-R	5'-CCGCTCGAGCCTCGCGGTAGTGGCTG-3'
**Mutagenesis**	
FOS-T232A-F	5'-CCAGAGGTTGCCGCCCCGGAGTCTG-3'
FOS-T232A-R	5'-CAGACTCCGGGGCGGCAACCTCTGG-3'
FOS-T325A-F	5'-GAGCCCCTGTGCGCTCCGGTGGTCA-3'
FOS-T325A-R	5'-TGACCACCGGAGCGCACAGGGGCTC-3'
FOS-T331A-F	5'-GGTGGTCACCTGTGCTCCCAGCTGCAC-3'
FOS-T331A-R	5'-GTGCAGCTGGGAGCACAGGTGACCACC-3'
FOS-S374A-F	5'-GACTCGCTCAGCGCACCCACGCTGC-3'
FOS-S374A-R	5'-GCAGCGTGGGTGCGCTGAGCGAGTC-3'
BRAF-A1799T-F	5'-ATTTTGGTCTAGCTACAGTGAAATCTCGATGGAGTGG-3'
BRAF-A1799T-R	5'-CCACTCCATCGAGATTTCACTGTAGCTAGACCAAAAT-3'
TERT-C228T-F	5'-GGCCCAGCCCCTTCCGGGCCCTC-3'
TERT-C228T-R	5'-GAGGGCCCGGAAGGGGCTGGGCC-3'
TERT-C250T-F	5'-CCGTCCCGACCCCTTCCGGGTCC-3'
TERT-C250T-R	5'-GGACCCGGAAGGGGTCGGGACGG-3'
GABPB-FOS-binding-mut-F	5'-GGATGCTGGGAGCTAGACTCACTCGCACA-3'
GABPB-FOS-binding-mut-R	5'-TGTGCGAGTGAGTCTAGCTCCCAGCATCC-3'
GABPB-MYC-binding-mut-F	5'-CTCACTCGCACACGATGTGTCCCTCCG-3'
GABPB-MYC-binding-mut-R	5'-CGGAGGGACACATCGTGTGCGAGTGAG-3'
**ChIP assay**	
TERT-F	5'-GGATTCGCGGGCACAGAC-3'
TERT-R	5'-GGGAGCGCGCGGCATCG-3'
GABPB-F	5'-AAAGATTCCGCACTCTCCGT-3'
GABPB-R	5'-AATCCCCACCGAAAAGTCCC-3'
